# Assessment of Healthcare Workers’ Preparedness for Managing Infectious Disease Outbreaks in Taif City, Saudi Arabia

**DOI:** 10.3390/healthcare13131494

**Published:** 2025-06-23

**Authors:** Ibtisam Qazi, Sultan S. Althobaiti, Manal M. Darwish, Yusuf S. Althobaiti, Abdullah S. Alzahrani, Waleed A. Mazi, Sameer Y. Awaji

**Affiliations:** 1 Department of Family and Community Medicine, College of Medicine, Jouf University, Sakaka 72388, Saudi Arabia; mdarwish@ju.edu.sa; 2 Public Health Department, Infection Prevention and Control Administration, Taif MOH Branch, Taif 21944, Saudi Arabia; susalthobaiti@moh.gov.sa (S.S.A.); syawaji@moh.gov.sa (S.Y.A.); 3 Department of Pharmacology and Toxicology, College of Pharmacy, Taif University, P.O. Box 11099, Taif 21944, Saudi Arabia; ys.althobaiti@tu.edu.sa; 4 Addiction and Neuroscience Research Unit, Health Science Campus, Taif University, P.O. Box 11099, Taif 21944, Saudi Arabia; 5 Population Health Management, Taif MOH Branch, Taif 21944, Saudi Arabia; aalzahrani176@moh.gov.sa; 6 Taif Health Cluster for Infection Prevention and Control, Infection Prevention and Control Director, Taif 21944, Saudi Arabia; wmazi@moh.gov.sa

**Keywords:** infectious diseases, healthcare workers, preparedness, outbreak management, Taif city

## Abstract

**Background and Objectives**: Infectious disease outbreaks are a major challenge for public health systems worldwide, especially for healthcare workers (HCWs). Taif city, in Saudi Arabia, has a high population density and is a tourist destination, which puts it at a high risk of infectious disease outbreaks. Despite its geographical importance, no previous study has been conducted that focuses on assessing the preparedness of healthcare workers in Taif city for managing infectious disease outbreaks. Therefore, we aimed to assess the overall level of preparedness among HCWs in healthcare facilities across Taif city and identify the challenges they face when managing infectious disease outbreaks. **Materials and Methods**: We conducted a cross-sectional study from October to December 2024 among 294 healthcare workers, using a structured questionnaire. We assessed the sociodemographic characteristics, infection prevention and control (IPC) training received by HCWs, the level of preparedness for managing infectious disease outbreaks, and their level of knowledge (low, moderate, or high). The association between sociodemographic characteristics and knowledge from having received IPC training and the level of preparedness was assessed using binary logistic regression. A *p*-value of ≤ 0.05 was considered as significant. **Results**: Around 31.7% of the participants were aged 31–40 years, with 59.2% of them being female. Among the HCWs we assessed, 44.6% were nurses and 31.3% of the HCWs were from hospitals with a bed capacity of over 500. Only 16.3% of HCWs felt fully prepared on a personal level and only 20.7% believed their facility was fully prepared for managing an outbreak. A low level of knowledge was reported among 71.8% of the participants. The odds of having received IPC training were significantly higher among HCWs aged 41–50 years (AOR = 15.7; 95% CI = 4.26–58.1), for those working in the inpatient department (AOR = 6.3; 95% CI = 1.46–27.05), and for those with a moderate level of knowledge (AOR = 0.12; 95% CI = 0.03–0.5). The odds of being fully prepared for an infectious disease outbreak were significantly higher for males (AOR = 2.58; 95% CI = 1.18–5.63) and those working in the in-patient department (AOR = 6.87; 95% CI = 1.7–27.8) and significantly lower for those with a low level of knowledge (AOR = 0.19; 95% CI = 0.06–0.61). **Conclusion**: Even though many HCWs have undergone IPC training, our findings highlight gaps in both knowledge and overall preparedness among healthcare workers in Taif city. Regular refresher courses, improved resource allocation, and implementing scenario-based emergency drills may help in improving the overall knowledge and preparedness of HCWs.

## 1. Introduction

Infectious disease outbreaks are a major challenge for public health systems worldwide, especially for healthcare workers (HCWs) [1,2]. Managing the COVID-19 pandemic was challenging for health systems globally, exposing many vulnerabilities within these systems, with HCWs being particularly at risk [3]. Since HCWs play an essential role in public health management, their readiness for managing outbreaks is essential for ensuring effective patient care and their own safety [4,5]. An adequate preparedness plan for responding to an emergency, with sufficient infrastructure support, properly trained personnel familiar, with specified roles and responsibilities, and with the necessary equipment that would be required in case of an outbreak, are essential for managing any outbreak [6]. These measures help in minimizing the impacts of an outbreak, playing a vital role in limiting spread and managing the disease burden [7].

A study conducted to assess the knowledge and preparedness of HCWs during the COVID-19 pandemic in Libya found that only 7% of the total number of participants were trained to manage COVID-19 cases. Out of these, 47.3% of doctors and 54.7% of nurses had received training on the proper use of PPE and only 20.6% doctors and 26.3% nurses felt that they were prepared for handling the pandemic [8]. A similar study conducted in Australia found that 77.8% of infectious disease (ID) physicians and 78.7% of infection control professionals (ICPs) had a good or very good level of knowledge about COVID-19. Around 66.7% of infectious disease (ID) physicians and 75% of infection control professionals (ICPs) had received training on PPE use. Almost 96% of ICPs and 73.3% of ID physicians felt moderately or extremely prepared for managing COVID-19 cases. A similar study from Saudi Arabia assessing healthcare workers readiness reported that 100% of HCWs kept a check on whether adequate numbers of PPE were available, with 83.4% reporting that they also checked alternate sources of PPE, thus contributing to the overall level of preparedness [9].

Studies assessing HCWs level of knowledge and preparedness during the COVID-19 pandemic have revealed wide-ranging levels of awareness and preparedness. However, very few studies have evaluated the level of knowledge and preparedness of HCWs after the pandemic, especially in the Middle Eastern region and, more specifically, in Saudi Arabia. Taif city is the sixth largest city in Saudi Arabia and is a popular destination for tourists and pilgrims [5,6]. Taif has a high population density, ranked 5th in the kingdom’s population census, and any outbreak could have a significant impact on the healthcare system [9,10]. The influx of tourists and pilgrims from all over the world increases the risk of infectious disease outbreaks [3]. The city has an extensive healthcare infrastructure, including 25 hospitals, 106 primary healthcare centers, and a large number of private healthcare facilities [7,8]. Despite its geographical importance as a tourist destination and stopover for pilgrims, to our knowledge, no previous study has been conducted that focuses on assessing the preparedness of healthcare workers in Taif city for managing infectious disease outbreaks [1,2]. Thus, our study fills an important gap in literature by focusing on two primary objectives: first, to assess the overall level of preparedness among HCWs in healthcare facilities across Taif city; and, second, to identify the challenges they face when managing infectious disease outbreaks. By identifying gaps and challenges, we hope to offer insights that can be used for improving preparedness, planning future studies, developing policy recommendations, and guiding resource allocation [10,11].

## 2. Materials and Methods

We conducted a cross-sectional study at all the private and governmental healthcare facilities, including hospitals and primary healthcare centers, in Taif city, from October to December 2024. HCWs from all these facilities were invited to participate in this study. Ethical approval for this study was obtained from the Taif Health Cluster (2024-E-77). We used data from a study that assessed the healthcare knowledge, preparedness, and experiences of health professionals managing COVID-19 in Australian healthcare settings for calculating the sample size [12]. The sample size was calculated using the WHO sample size calculator, using the following assumptions: a confidence level of 95%, a margin of error of 5%, and a population proportion of 78.7%. The required sample size was calculated to be 260 and we added 10% to account for incomplete questionnaires, thus the required sample size was 286 [12].

We invited all eligible HCWs involved in patient care, working in all healthcare facilities, both private and governmental, in Taif city, to participate in our study and excluded all those who did not give their consent. The questionnaires were distributed online to all the HCWs, using a convenience sampling technique. A structured questionnaire was developed to assess infectious disease preparedness among HCWs, using previously validated questions from the National Guide for Auditors in Infection Control from the Ministry of Health, Saudi Arabia, and the outbreak preparedness checklist fact sheet from the Australian Government Department of Health and Aged Care. The questionnaire was piloted among the healthcare workers to assess the comprehensibility and clarity of the questions. No changes were required to the questionnaire before it was distributed online for data collection [13,14]. The questionnaire was distributed in English, as all the HCWs were familiar with the language. The questionnaire consisted of three sections. Section A comprised questions related to sociodemographic characteristics, such as age, gender, job description, type of health facility, and working unit. Section B consisted of questions related to their knowledge about infectious disease and the color codes used to alert staff about infection control precautions (such as blue for airborne, red for droplet, and green for contact), and Section C assessed the infection prevention and control (IPC) training received by HCWs, the level of preparedness for managing infectious disease outbreaks [13], and the challenges faced during outbreak preparedness [13,14]. Both Sections B and C had questions derived and modified from the National Guide for Auditors in Infection Control from the Ministry of Health, Saudi Arabia, and the outbreak preparedness checklist fact sheet from the Australian Government Department of Health and Aged Care.

The data was analyzed using SPSS version 23. Descriptive statistics, including frequencies and percentages, were used to summarize the categorical variables, such as sociodemographic characteristics, the training received by HCWs, the level of preparedness for managing infectious disease outbreaks, and the challenges faced during outbreak preparedness. The knowledge section of the questionnaire (Section B) had 11 questions to assess the HCWs’ understanding of infectious disease preparedness. The participants were given scores out of 11 and we used Bloom’s cutoffs for knowledge to categorize these scores. The participants who had a score of less than 7 (<60%) were categorized as having a low level of knowledge, those with scores between 7 and 8 (60–79%) were considered to have a moderate level of knowledge, and those scoring between 8 and 11 (80–100%) were considered to have a high level of knowledge [15].

The association between the sociodemographic characteristics and knowledge from having received IPC training and the level of preparedness was assessed using binary logistic regression for the univariate and multivariate analysis, respectively. Our outcome variables were having received IPC training and the level of preparedness. We assessed having received IPC training through two responses: yes or no. The level of preparedness was assessed by asking the HCWs whether they were fully prepared or not fully prepared. Crude and adjusted odds ratios, with 95% confidence intervals, were reported. Any variable with a *p*-value ≤ 0.25 in the univariate analysis was considered eligible for multivariate analysis.

The variables used in the univariate analysis were age, gender, job description, type of health facility, working unit, and level of knowledge. Age was grouped into 4 categories: from 20 to 30 years, 31–40 years, 41–50 years, and 51 years and above. We used 20–30 years as the reference category. For gender, we used females as the reference category. Job description was grouped into five categories, namely doctors, dentists, nurses, IPC team members, and others, which included laboratory technicians, pharmacists, and physiotherapists. We used doctors as our reference category. Healthcare facilities were placed into five categories as well, namely primary healthcare centers and hospitals with a 50–200 bed capacity, 201–350 bed capacity, 351–500 bed capacity, and hospitals with a more than 500 bed capacity. We used hospitals with a more than 500 bed capacity as our reference category. Working units were placed into five categories, namely emergency room (E.R.) and critical care units, out-patient departments (OPDs), in-patient departments (IPDs), infection control unit, and other working units. We used the emergency room (E.R.) and critical care units as our reference category. The level of knowledge was placed into three categories, namely low, moderate, and high levels of knowledge. We used a high level of knowledge as our reference category.

We included all the variables that had a *p*-value of ≤ 0.25 in the univariate analysis in the final multivariate analysis. For the having received IPC training, the variables that had a *p*-value of ≤ 0.25 were age, gender, job description, working unit, and level of knowledge. These were included in the final multivariate model. The type of health facility was insignificant at the univariate level for the having received IPC training variable and, hence, was not used in the final multivariate analysis. The final multivariate model for the having received IPC training was adjusted for age, gender, job description, working unit, and level of knowledge.

For the level of preparedness variable, the variables with a *p*-value of ≤ 0.25 at the univariate level were age, gender, job description, type of health facility, working unit, and level of knowledge. The final multivariate model for the level of preparedness variable was adjusted for age, gender, job description, type of health facility, working unit, and level of knowledge. In addition to statistical significance in the univariate analysis, clinical relevance and findings from previous literature were also considered during model selection to ensure meaningful interpretation. A *p*-value of ≤ 0.05 was considered to be significant.

## 3. Results

A total of 294 HCWs from various facilities in Taif city had filled in the questionnaire by the time we stopped the data collection. We had a response rate exceeding 100% and decided to include all the questionnaires in the analysis. Around 31.7% of the participants were aged 31–40 years, followed by 30.3% aged 41–50 years, with 59.2% of the participants being female. When looking at the job descriptions, we found that 44.6% were nurses, which was the largest group of HCWs, and 31.3% of the HCWs were from hospitals with a bed capacity of over 500 (Table 1).

When assessing the preparedness of the HCWs for managing infectious disease outbreaks, 85.7% reported having received training in infection prevention and control (IPC) measures, with 44.9% having had one refresher training. When evaluating personal preparedness, only 16.3% of HCWs felt fully prepared, while 83.7% felt they were not fully prepared for managing an outbreak. At the facility level, 20.7% believed their facility was fully prepared, while 79.2% believed their facility had undertaken little preparations for managing an outbreak (Table 2).

The knowledge levels varied significantly among the participants, with only 51% correctly identifying the causes of infectious diseases and only 46.6% correctly answering questions related to the transmission routes of various diseases. Around 73.5% of HCWs were knowledgeable about the correct color codes for airborne precautions, but only 26.5% answered correctly about the correct color codes for droplet and contact precautions. Knowledge about the correct personal protective equipment (PPE) ranged from 37.4% (for droplet precautions) to 60.5% (for contact precautions). We summarized the knowledge scores for the HCWs with scores ranging from 0 to 11, with a mean knowledge score of 4.9 ± 2.98. Almost 71.8% of the participants scored between 0 and 6, signifying a low level of knowledge about infectious disease and the color codes used in infection control precautions (Table 3).

For the IPC training, except for healthcare facilities, all the other sociodemographic variables and the level of knowledge were significant at the univariate level and were included in the final multivariate model. For the level of preparedness, all the sociodemographic variables and the level of knowledge were significant at the univariate level and were included in the final multivariate model. We reported the adjusted odds ratios after adjusting for potential covariates in the multivariate analysis. The odds of having received IPC training were significantly higher among the HCWs aged 41–50 years (AOR = 15.7; 95% CI = 4.26–58.1), for those working in the inpatient department (AOR = 6.3; 95% CI = 1.46–27.05), and for those with a moderate level of knowledge (AOR = 0.12; 95% CI = 0.03–0.5). The odds of being fully prepared for infectious disease outbreaks were significantly higher for males (AOR = 2.58; 95% CI = 1.18–5.63) and for those working in the inpatient department (AOR = 6.87; 95% CI = 1.7–27.8) and significantly lower for IPC team members (AOR = 0.13; 95% CI = 0.03–0.53) and for those with a low level of knowledge (AOR = 0.19; 95% CI = 0.06–0.61) (Table 4).

Many challenges were reported by the HCWs in regard to their overall preparedness. Around 24.2% of HCWs reported a lack of training, 19.9% reported a deficiency in terms of adequate numbers of staff, 17.5% reported an absence of emergency drills, 12.8% reported a shortage of equipment, 15.7% reported the absence of guidelines, and 10% reported deficient knowledge, among the challenges they faced in regard to outbreak preparedness. Almost 47.2% of HCWs highlighted the need for improved training, while 24% said they would like to enhance their knowledge about infection control practices and 28.8% said they would like training on better utilization of the equipment.

## 4. Discussion

Despite the availability of training programs and resources, the results from our study highlight critical gaps in knowledge and preparedness related to infectious disease outbreaks among HCWs in Taif city. While assessing the preparedness of HCWs for managing infectious disease outbreaks, 85.7% reported receiving training on infection prevention and control (IPC) measures and 83.7% felt they were not fully prepared for managing an outbreak. Almost 71.8% of participants scored between 0 and 6, signifying a low level of knowledge about infectious disease and the color codes used in infection control precautions. The knowledge scores further illustrate these gaps, with a majority of participants having a low level of knowledge about infectious disease and infection control precautions. This low level of knowledge was demonstrated despite 85.7% of the participants having received infection prevention and control (IPC) training. This could be due to the fact that only 13.9% of participants had refresher training, which is vital for updating and retaining knowledge and information on the relevant procedures. According to a survey conducted in Botswana, only 51.3% of healthcare workers at Lobatse District Health Management Team facilities had sufficient understanding of infection prevention and control (IPC), and 50.6% engaged in safe IPC practices [16]. In Nigeria, a study found that more than 90% of healthcare workers (HCWs) had adequate knowledge about COVID-19, but only 72.3% were aware of its incubation period, indicating gaps in critical information that could affect infection control practices [10]. A similar study from Yemen reported that HCWs perceived their level of readiness in relation to the prior training that they had received in crisis management. Overall, only 39.2% of HCWs had received any form of training on crisis management [17].

Deficiencies in training can lead to HCWs being reluctant to work during disease outbreaks. A study conducted in the southern region of Saudi Arabia, by Sultan et al., evaluated emergency healthcare providers’ perceptions of preparedness and their willingness to work during disasters and public health emergencies. Their findings revealed that the willingness to work varied significantly depending on the type of event, with only 43.56% willing to work during SARS/COVID-19 pandemics, and even lower rates for other high-risk scenarios, such as chemical or biological events and nuclear incidents. This study highlighted that a lack of confidence and inadequate assurance of safety for healthcare workers and their families were major deterrents to their willingness to work. These results are comparable to our findings, where only 16.3% of HCWs reported feeling fully prepared on a personal level to manage disease outbreaks, and where low knowledge scores were prevalent despite high reported rates of IPC training. Both studies suggest that emotional readiness and perceived safety play critical roles, alongside technical training, in shaping healthcare workers’ preparedness and willingness to respond [18]. In Yemen, only 66% of HCWs were willing to work during an influenza epidemic. Ensuring workplace safety and having previous experience of participating in an outbreak (AOR 1.52, 95% CI = 1.058 to 2.207) were factors that influenced participation by HCWs. In the USA, more than 65% of healthcare workers were willing to work during a disaster as compared to only 54% willing to work during an influenza epidemic, 41% during a chemical exposure incident, and 39% during a radiation exposure incident. Being familiar with an emergency plan increased the chances of these HCWs working during these situations [17,19]. When evaluating personal preparedness, we found that only 16.3% of HCWs felt fully prepared and only 20.7% believed their facility was fully prepared for managing a disease outbreak. A multi-center research project carried out in the USA, in large metropolitan facilities, including hospitals with special pathogens teams, found that these facilities were the main predictors of preparedness [20]. According to research conducted in Ethiopia, healthcare workers who took part in IPC training had a higher likelihood of having sufficient IPC knowledge than those who did not (AOR = 2.2, 95% CI = 1.01–4.75). Furthermore, the knowledge scores were significantly impacted by having access to IPC guidelines (AOR = 3.65, 95% CI = 1.26–10.54) and for those who had guidelines available [21]. Similarly in our study, the odds of having received IPC training were significantly lower among those who had a low level of knowledge (AOR = 0.12; 95% CI = 0.03–0.5). In Nigeria, nearly all HCWs surveyed felt their facilities were insufficiently equipped to respond to disease outbreaks, with preparedness and knowledge largely limited to basic universal precautions and little mention of training or emergency drills [22]. In Libya, only about 20.6% of doctors and 26.3% of nurses felt personally prepared for the COVID-19 outbreak, and less than 7% had received training on case management [8]. Our finding that only 16.3% of HCWs felt fully prepared aligns with outcomes in other low- and middle-income countries. In Libya, only 20.6% of doctors and 26.3% of nurses felt prepared to handle the COVID-19 outbreak, with fewer than 7% trained in case management [8]. In Nigeria, healthcare workers widely reported that their facilities lacked adequate resources and preparedness measures, such as drills or formal training [22]. Conversely, higher levels of preparedness have been observed in high-income countries. For instance, a study in Australia reported that 96% of infection control professionals and 73.3% of infectious disease physicians felt moderately or extremely prepared, supported by widespread PPE training [12]. These contrasting findings highlight the critical impact of national infrastructure, emergency protocols, and ongoing training systems in shaping outbreak preparedness.

Several studies have emphasized the importance of professional development and systematic training to improve HCWs’ preparedness [23,24,25]. In Taif city, gaps in emergency responses and staffing among nurses were observed in regard to disaster management [26]. Similar results were reported from a study carried out in Ghana, where preparedness levels in a majority of the facilities were low, despite having received IPC training [27]. These data emphasize the critical need for more systematic preparedness training and emergency simulation exercises. Variations in the level according to which healthcare workers are prepared relative to one another and relative to the relevant systems at the facility level may indicate unequal access to training opportunities and the unequal distribution of resources. Such discrepancies are most probably responsible for the subjective perceived readiness of HCWs. These findings indicate the demand for systematic and ongoing training programs to close gaps in knowledge [28]. The Kingdom of Saudi Arabia has developed solid procedures through the Ministry of Health’s Healthcare-Associated Outbreak Management Manual, through which it offers guidance on outbreak control in healthcare settings. Among them are the early detection of cases, immediate notification, the development of workforce training programs, and coordinated logistical support [28]. The document also addresses systemic challenges in terms of impact and supply constraints, which may limit the ability to respond effectively to disease outbreaks and require swift administrative interventions. It has set forth well-articulated protocols that proactive countries have embraced in regard to increasing preparedness for disease outbreaks; however, empirical data, especially survey-based evidence, is lacking regarding self-reported readiness or perceptions of facility preparedness among healthcare workers. This shows that preparedness might be standardized, but differs between institutions, due to differences in the accessed training, availability of resources, and operational capacities.

Based on the findings of our study, several essential recommendations may be developed to better prepare healthcare workers (HCWs) in Taif city to deal with disease outbreaks. We found low levels of preparedness for managing a disease outbreak, despite large numbers of HCWs having received IPC training. This could be due to many factors, such as outdated and irrelevant training materials and techniques and infrequent refresher training. Training programs need to be enhanced with regular refresher courses on the identification of an outbreak, approaches on how to respond, and infection prevention and control (IPC) measures. Regular educational programs can help equip HCWs by increasing their awareness and readiness to deal effectively with new public health challenges. In addition to this, resource allocation must be optimized to provide adequate staffing levels and ensure the ongoing availability of essential personal protective equipment (PPE) and medical supplies. Enhancing supply chain management and having emergency stockpiles can help enhance the healthcare system’s ability to respond to outbreaks effectively.

Considering the scarcity of data about outbreak readiness from the Middle Eastern region and especially Saudi Arabia, our study has helped address this knowledge gap. Since all the health facilities across Taif city were asked to participate, the results represent valuable regional findings. Another major strength is the application of a structured questionnaire derived from the literature, which allowed for thorough data collection on HCWs’ knowledge, training experience, and preparedness. However, the findings may not have the potential to be generalized to other sites across Saudi Arabia, due to the wide geographical variation and differences in healthcare infrastructure, policies, and levels of outbreak preparedness. The use of self-reported questionnaires can introduce recall bias or social desirability bias, which can influence the validity of responses. Despite being significant, our confidence intervals were wide, which may indicate high variability within our dataset, thus affecting the precision of the point estimate. Moreover, the use of convenience sampling may have introduced selection bias, affecting the diversity of the respondents and potentially limiting the representativeness of the findings. Self-reported responses may also carry the risk of either overestimation or underreporting of preparedness and training experiences. Despite these limitations, the inclusion of participants from both public and private healthcare sectors across Taif city strengthens the generalizability of the findings within the region. Furthermore, the application of a structured and pre-tested questionnaire supports the reliability of the data collection process. We recommend further studies in this area involving a more homogenous population to counter this effect.

## 5. Conclusions

This study identifies crucial gaps in HCWs’ knowledge and preparedness in Taif city, emphasizing the need for targeted interventions. While IPC training is widely available, its impact on knowledge retention is limited, as a majority of the participants demonstrated poor knowledge of infection control practices. These findings indicate the need for systematic and ongoing training programs to close gaps in knowledge. We recommend structured and interactive training approaches, periodic refresher courses, adequate resource allocation, and scenario-based emergency drills, which can help enhance preparedness. These improvements can help the healthcare system be better equipped to manage future infectious disease outbreaks.

## Figures and Tables

**Table 1:** Sociodemographic characteristics of healthcare workers (HCWs) (n=294).

Variables	Categories	Frequency (n)	Percentage (%)
Age	20–30 years	31	10.5
	31–40 years	109	37.1
	41–50 years	89	30.3
	51 years and above	65	22.1
Gender	Male	120	40.8
	Female	174	59.2
Job description	Doctor	24	8.2
	Dentist	26	8.8
	Nurse	131	44.6
	IPC team member	48	16.3
	Others *	65	22.1
Healthcare facility	Primary healthcare center	37	12.6
	Hospital with 50–200 bed capacity	77	26.2
	Hospital with 201–350 bed capacity	9	3.1
	Hospital with 351–500 bed capacity	79	26.9
	Hospital More than 500 bed capacity	92	31.3
Working unit	Emergency room (E.R.) and critical care units	55	18.7
	Out-patient department (OPD)	54	18.4
	Inpatient department (IPD)	55	18.7
	Infection control unit	56	19
	Other working units +	74	25.2

* Includes laboratory technicians, pharmacists, and physiotherapists. + Other working units include radiology, pharmacy, and physiotherapy.

**Table 2:** Preparedness of HCWs for managing infectious disease outbreaks (n = 294).

Variables	Categories	Frequency (n)	Percentage (%)
Received training on IPC measures	No	42	14.3
	Yes	252	85.7
Received refresher training on IPC measures	Never	41	13.9
	Once	132	44.9
	Twice	77	26.2
	More than two times	44	15
Emergency management and outbreak identification training	No	56	19
	Yes	238	81
Communication of procedure/policy for outbreak preparedness	No	48	16.3
	Yes	246	83.7
Presence of up-to-date outbreak management plan	No	28	9.5
	Yes	217	73.8
	Not sure	49	16.7
Testing of outbreak management plan through scenario-based activities	No	32	10.9
	Yes	212	72.1
	Not sure	50	17
Received any training and education regarding the outbreak management plan in your organization	No	66	22.4
	Yes	228	77.6
Number of times refresher training on this plan was given	Never	66	22.4
	Once	103	35
	Twice	100	34
	More than two times	25	8.5
Knowledge of who to inform if you identify a suspected infection that can cause an outbreak	No	30	10.2
	Yes	264	89.8
Knowledge about lead person for managing the outbreak in your organization	No	32	10.9
	Yes	262	89.1
Familiarity with the team members that manage outbreaks in your organization	No	39	13.3
	Yes	255	86.7
Clear roles and responsibilities of the outbreak management team	No	47	16
	Yes	247	84
Plan for communicating with staff, family members, and other service providers	No	22	7.5
	Yes	226	76.9
	Not sure	46	15.6
Easy availability of up-to-date contact details for the outbreak team	No	42	14.3
	Yes	252	85.7
Presence of enough qualified staff to manage outbreaks if they happen	No	25	8.5
	Yes	221	75.2
	Not sure	48	16.3
Presence of adequate supplies of PPE (gloves, masks, hand hygiene kits, and environmental cleaning supplies)	No	10	3.4
	Yes	243	82.7
	Not sure	41	13.9
Presence of backup resources of PPE (gloves, masks, hand hygiene kits, environmental cleaning supplies)	No	36	12.2
	Yes	258	87.8
Ever participated in the management of an outbreak	No	86	29.3
	Yes	208	70.7
Do you feel that you are prepared to participate in managing an outbreak if it happens?	Not fully prepared	246	83.7
	Fully prepared	48	16.3
Do you feel that your healthcare facility is prepared to participate in managing an outbreak if it happens?	Not fully prepared	233	79.2
	Fully prepared	61	20.7

**Table 3:** Knowledge scores for HCWs about infectious disease outbreak preparedness (n = 294).

Knowledge Scores	Frequency (n)	Percentage (%)	Level of Knowledge
Between 0 and 6	211	71.8	Low
Between 7 and 8	26	8.9	Moderate
Between 9 and 11	57	19.3	Good

**Table 4:** Binary logistic regression analysis of factors associated infection prevention and control (IPC) training and level of preparedness of HCWs (n = 294).

Variables	Categories	Received IPC Training		Level of Preparedness	
		Adjusted OR (95%CI)	*p*-Value	Adjusted OR (95%CI)	*p*-Value
Age	20–30 years	Ref.		Ref.	
	31–40 years	7.7 (2.6–22.9)	0.0001	1.24 (0.37–4.18)	0.72
	41–50 years	15.7 (4.26–58.1)	0.0001	1.16 (0.31–4.29)	0.83
	51 years and above	14.6 (3.51–60.8)	0.0001	0.7 (0.16–3.11)	0.65
Gender	Female	Ref.		Ref.	
	Male	1.12 (0.49–2.56)	0.8	2.58 (1.18–5.63)	0.02
Job description	Doctor	Ref.		Ref.	
	Dentist	1.81 (0.14–22.6)	0.65	0.14 (0.02–0.98)	0.05
	Nurse	1.09 (0.24–5.06)	0.91	0.39 (0.13–1.25)	0.12
	IPC team member	0.88 (0.14–5.38)	0.89	0.13 (0.03–0.53)	0.005
	Others	0.64 (0.13–3.23)	0.58	0.16 (0.04–0.66)	0.01
Health facility	Primary healthcare center	--		1.42 (0.43–4.63)	0.56
	Hospital with 50–200 bed capacity	--		0.57 (0.19–1.72)	0.32
	Hospital with 201–350 bed capacity	--		0.45 (0.04–4.58)	0.5
	Hospital with 351–500 bed capacity	--		0.46 (0.14–1.48)	0.19
	Hospital with more than 500 bed capacity	--		Ref.	
Working unit	Emergency room (E.R.) and critical care units	Ref.		Ref.	
	Out-patient department (OPD)	2.44 (0.74–8.030)	0.14	1.8 (0.38–8.56)	0.5
	Inpatient department (IPD)	6.30 (1.46–27.05)	0.01	6.87 (1.7–27.8)	0.007
	Infection control unit	5.99 (1.18–30.2)	0.03	5.51 (1.36–22.22)	0.02
	Other working units	1.8 (0.6–5.3)	0.28	4.08 (1.11–15.04)	0.03
Level of knowledge	High level	Ref.		Ref.	
	Moderate level	0.12 (0.03–0.5)	0.003	0.33 (0.08–1.3)	0.11
	Low level	0.55 (0.16–1.91)	0.35	0.19 (0.06–0.61)	0.005

## Data Availability

The raw data supporting the conclusion of this article will be made available by the authors upon request.
